# Supplementary material for: MetaQTL: a package of new computational methods for the meta-analysis of QTL mapping experiments
Source: BMC Bioinformatics. 2007 Feb 8;8:49. doi: 10.1186/1471-2105-8-49 (PMC1808479; doi:10.1186/1471-2105-8-49)
Supplement: Additional File 7 — MetaQTL Package : jar file and tutorial. This Zip archive contains both the MetaQTL JAR file and the files of the tutorial. [file 1471-2105-8-49-S7.zip › org.inra.metaqtl/doc/org/metaqtl/CMarkerSequence.html]

CMarkerSequence


|  |  |  |
| --- | --- | --- |
| **Overview** \| **Package** \| **Class** \| **Use** \| **Tree** \| **Deprecated** \| **Index** \| **Help** | |  |
| **PREV CLASS**   **NEXT CLASS** | **FRAMES**    **NO FRAMES**     **All Classes** |  |
| SUMMARY: NESTED \| FIELD \| CONSTR \| METHOD | DETAIL: FIELD \| CONSTR \| METHOD |  |


---


## org.metaqtl Class CMarkerSequence

```
java.lang.Object
  org.metaqtl.CMarkerSequence
```

---

``` public class CMarkerSequence extends java.lang.Object ```

A Common marker sequence between two ordered set of markers
is a sub-set of at least two markers which order is the same between
the two sets (may be reverse).
For example :
Set 1 : {a,b,c,d,e}
Set 2 : {a,b,e,d,c}
Then this two sets have two common sequences {a,b} and {d,e}.
Hereafter the term 'Common Marker Sequence' is used as well as for
a single commom sequence than for the set of common sequences for
a same comparison between two ordered sets.

---

| **Field Summary** | |
| --- | --- |
| `int[]` | `css`             The sizes of the common sequences |
| `boolean[]` | `frames`             The frame of the common marker sequences |
| `int[][]` | `idx1`             The indices of the markers on the first chromosome for the common sequences. |
| `int[][]` | `idx2`             The indices of the marker on the second chromosome for the common sequences. |
| `boolean[]` | `incms` |
| `int[][]` | `mcidx`             The common marker indices on the chromosomes. |
| `int` | `ncs`             The number of common sequences. |
| `int` | `nmc`             The number of common marker between chromosomes |


**Constructor Summary**

`CMarkerSequence(int[][] mcidx,
int nmc)`
  
          Initiates a new Common marker sequence from the
tow arrays of common marker indices
`mcidx[0..1][0..nmc-1]`.


| **Method Summary** | |
| --- | --- |
| `int[]` | `getCSIdx(int i, int m1)` |
| `int[]` | `getLeftCSMarker(int cs1, int m1x)`             Returns the indices of the marker at the left of the given one. |
| `int` | `getMarkerIdx(int i, int cidx, int midx)` |
| `int[]` | `getRightCSMarker(int cs2, int m2x)` |
| `boolean` | `haveSameFrame(int cs1, int cs2)` |

**Methods inherited from class java.lang.Object**

`clone, equals, finalize, getClass, hashCode, notify, notifyAll, toString, wait, wait, wait`

**Field Detail**

### nmc

```
public int nmc
```

:   The number of common marker between chromosomes

---


### mcidx

```
public int[][] mcidx
```

:   The common marker indices on the chromosomes.

---


### incms

```
public boolean[] incms
```

---


### ncs

```
public int ncs
```

:   The number of common sequences.

---


### css

```
public int[] css
```

:   The sizes of the common sequences

---


### frames

```
public boolean[] frames
```

:   The frame of the common marker sequences

---


### idx1

```
public int[][] idx1
```

:   The indices of the markers on the first
    chromosome for the common sequences.

---


### idx2

```
public int[][] idx2
```

:   The indices of the marker on the second
    chromosome for the common sequences.


**Constructor Detail**

### CMarkerSequence

```
public CMarkerSequence(int[][] mcidx,
                       int nmc)
```

:   Initiates a new Common marker sequence from the
    tow arrays of common marker indices
    `mcidx[0..1][0..nmc-1]`. This assumes
    that `mcidx` has been obtained by using
    one of the two orderd set as the reference.

    **Parameters:**: `mcidx` - the two arrays of common marker indices.: `nmc` - the number of common markers.


**Method Detail**

### getCSIdx

```
public int[] getCSIdx(int i,
                      int m1)
```

:   **Parameters:**: `m1` - the indice of the marker. **Returns:**: the indices of both the common sequence which contains the marker and the indice of the marker inside the sequence.

---


### getLeftCSMarker

```
public int[] getLeftCSMarker(int cs1,
                             int m1x)
```

:   Returns the indices of the marker at the left of the
    given one.

    :   **Parameters:**: `cs1` -: `m1x` - **Returns:**

---


### getRightCSMarker

```
public int[] getRightCSMarker(int cs2,
                              int m2x)
```

:   **Parameters:**: `cs2` -: `m2x` - **Returns:**

---


### getMarkerIdx

```
public int getMarkerIdx(int i,
                        int cidx,
                        int midx)
```

:   **Parameters:**: `i` -: `cs1` -: `m1x` - **Returns:**

---


### haveSameFrame

```
public boolean haveSameFrame(int cs1,
                             int cs2)
```

:   **Parameters:**: `cs1` -: `cs2` -


---


|  |  |  |
| --- | --- | --- |
| **Overview** \| **Package** \| **Class** \| **Use** \| **Tree** \| **Deprecated** \| **Index** \| **Help** | |  |
| **PREV CLASS**   **NEXT CLASS** | **FRAMES**    **NO FRAMES**     **All Classes** |  |
| SUMMARY: NESTED \| FIELD \| CONSTR \| METHOD | DETAIL: FIELD \| CONSTR \| METHOD |  |


---
